# Editorial: Chronic subdural hematoma (CSDH) - a well-known unknown

**DOI:** 10.3389/fneur.2024.1461117

**Published:** 2024-07-30

**Authors:** Milan Lepić, Hiroki Sato

**Affiliations:** ^1^Medical Faculty of the Military Medical Academy, University of Defence, Belgrade, Serbia; ^2^Clinic for Neurosurgery, Military Medical Academy, Belgrade, Serbia; ^3^Department of Neurosurgery, Teikyo University Mizonokuchi Hospital, Kawasaki, Japan

**Keywords:** chronic subdural hematoma (CSDH), management, surgical treatment, endovascular treatment, adjuvant treatment options, reoccurrence, outcome

Chronic subdural hematoma (CSDH) is a common neurosurgical condition that primarily affects the elderly population, characterized by the accumulation of fluid between the dura mater and the arachnoid membrane of the brain. Despite being a well-known entity, CSDH remains a subject of controversy, from its name and origin to advanced therapeutic approaches. Unlike acute subdural hematomas, which typically result from significant head injury, CSDHs can develop insidiously, making their diagnosis and treatment challenging (1).

This condition is increasingly prevalent among the frail and elderly populations due to their common use of anticoagulant and antiplatelet therapy, and age-related vascular fragility. Additionally, the aging population is more prone to underlying chronic diseases that may exacerbate the formation of subdural hematomas. As the global population continues to age, the incidence of CSDH is expected to double over the next decade, presenting a growing public health concern (2).

Understanding the multifaceted nature of CSDH and its growing incidence in the elderly is crucial for developing effective management strategies and improving patient outcomes. Recent research has expanded our understanding of the factors influencing the recurrence, treatment, and outcomes of CSDH, offering new insights that can shape future clinical practices. This Research Topic presents 12 pivotal studies that delve into the nuances of CSDH understanding and management, each contributing valuable evidence and perspectives to the field, focusing on the current state of CSDH management and proposing innovative strategies for improving patient outcomes.

The study titled “*Effectiveness of tranexamic acid on chronic subdural hematoma recurrence: a meta-analysis and systematic review*” by Pan et al. conducted a meta-analysis comparing the effectiveness of tranexamic acid (TXA) in reducing CSDH recurrence rates. Their findings suggest that TXA, an antifibrinolytic agent, can significantly lower postoperative recurrence rates by 67% compared to standard neurosurgical treatment alone. Furthermore, the study found no significant differences in the incidence of thrombosis or mortality between TXA and control groups, with myocardial infarction being less frequent in the TXA group. These results highlight the potential of TXA as an adjuvant therapy for CSDH, providing high-level evidence to support its clinical use (Pan et al.).

In “*Preservation of the middle meningeal artery during unruptured aneurysm surgery: an independent risk factor for postoperative chronic subdural hematoma*,” by Kim investigated the association between the preservation of the anterior branch of the middle meningeal artery (MMA) during unruptured and surgery and the development of postoperative CSDH. The study revealed that MMA preservation, advanced age, and male sex are independent risk factors for CSDH following unruptured aneurysm clipping. These findings underscore the importance of considering MMA management during neurosurgical procedures and may guide surgical planning to mitigate postoperative CSDH risk (Kim).

The “*Advances in chronic subrural hematoma and membrane imaging*” by Chen et al. provided a comprehensive review of the current landscape of CSDH diagnosis and treatment, with a focus on advanced imaging techniques for assessing hematomas and subdural membranes. The authors discussed the potential role of MR and dual-energy CT imaging in predicting CSDH recurrence, surgical planning, and patient selection for MMA embolization treatment. As CSDH recurrence remains a significant challenge despite conventional management, the development of novel radiographic biomarkers to guide treatment decisions is a promising avenue for future research (Chen et al.).

A systematic review and meta-analysis titled “*Intraoperative irrigation of artificial cerebrospinal fluid and temperature of irrigation fluid for chronic subdural hematoma: a systematic review and meta-analysis*” by Huang et al. conducted comparing the efficacy of artificial cerebrospinal fluid (ACF) and normal saline (NS) as irrigation fluids during CSDH surgery. Their findings suggest that ACF may reduce postoperative recurrence rates by 47% compared to NS. Furthermore, they discovered that using irrigation fluid at body temperature could decrease recurrence rates by 64% compared to room temperature fluid. These results highlight the importance of optimizing intraoperative techniques to minimize CSDH recurrence (Huang et al.).

A “*Classification of subdural hematomas: proposal for a new system improving the ICD coding tools*” by Langlois et al. proposed a new classification system for subdural hematomas that captures the chronicity and etiology of the condition, factors that significantly impact management and prognosis. The current ICD coding system fails to adequately distinguish between acute and chronic subdural hematomas, hindering administrative, statistical, and research applications. The authors' proposed classification system offers a more comprehensive approach to categorizing subdural hematomas, which could lead to improved patient care and research outcomes (Langlois et al.).

Yang W. et al. investigated the correlation between skull density and CSDH progression in the study titled “*Predicting the progression of chronic subdural hematoma based on skull density*”. Their study revealed that lower minimum skull density, higher maximum skull density, and higher skull density difference were significantly associated with CSDH progression. The authors developed and validated a predictive model incorporating these factors, which could aid in early assessment of CSDH progression and guide treatment decisions (Yang W. et al.).

The study titled “*Case report: concurrent low-volume subdural hematoma and ipsilateral ischemic stroke presenting as capsular warning syndrome: a complex case with anticoagulation dilemma and dual pathology*” by Strahnen et al. presented a complex case report of a patient with concurrent low-volume subdural hematoma and ipsilateral ischemic stroke, highlighting the challenges of managing anticoagulation in such scenarios. The patient's presentation with capsular warning syndrome further complicates the clinical picture. This case underscores the need for a multidisciplinary approach and the development of tailored treatment strategies for patients with multiple comorbidities (Strahnen et al.).

In “*Middle meningeal artery embolization for chronic subdural hematoma: a systematic review*,” by Omura and Ishiguro conducted a systematic review of middle meningeal artery embolization (MMAE) for CSDH. Their analysis suggests that MMAE alone is as effective as evacuation surgery in reducing hematoma, although the effect is not immediate. Additionally, they found that combining MMAE with evacuation surgery results in lower recurrence rates compared to evacuation surgery alone. Given the safety profile of MMAE, the authors recommend considering this procedure for patients with CSDH, particularly those at high risk of recurrence (Omura and Ishiguro).

The study titled “*Effect of decreased platelets on postoperative recurrence of chronic subdural hematoma*” by Yagi et al. investigates the role of thrombocytopenia in the recurrence of CSDH post-surgery. This research highlights a crucial aspect of patient care, underscoring the need for meticulous perioperative management of platelet levels to minimize the risk of recurrence. The findings suggest that lower platelet counts may be a significant risk factor, prompting the need for targeted therapeutic strategies to mitigate this risk (Yagi et al.).

In “*Nontraumatic subdural hematoma in patients on hemodialysis with end-stage kidney disease: a systematic review and pooled analysis*,” by Yang L. et al. the authors examine the incidence and outcomes of non-traumatic subdural hematomas in a particularly vulnerable patient population. Patients with end-stage kidney disease (ESKD) on hemodialysis are at increased risk due to anticoagulation and uremic platelet dysfunction. This comprehensive review and pooled analysis provide critical insights into the management of these patients, emphasizing the importance of tailored therapeutic approaches to improve outcomes (Yang L. et al.).

The “*Success of conservative therapy for chronic subdural hematoma patients: a systematic review*” by Foppen et al. explores the viability of non-surgical management of CSDH. This systematic review synthesizes data from multiple studies to evaluate the effectiveness of conservative treatments such as corticosteroids and mannitol. The findings support conservative therapy as a feasible option for certain patient cohorts, particularly those with mild symptoms or significant surgical risk, potentially reducing the need for invasive procedures (Foppen et al.).

Finally, the population-based study “*Incidence, therapy, and outcome in the management of chronic subdural hematoma in Switzerland: a population-based multi-center cohort study*” by El Rahal et al. provides a comprehensive overview of the epidemiology and management practices of CSDH across multiple centers in Switzerland. This cohort study offers valuable epidemiological data and compares the outcomes of various therapeutic interventions, thereby contributing to the optimization of treatment protocols and healthcare policies (El Rahal et al.).

Collectively, these studies advance our understanding of CSDH by identifying key risk factors, evaluating diverse patient populations, and comparing therapeutic approaches. They underscore the importance of individualized patient care and the need for continued research to refine management strategies. The exact etiology and pathophysiology of CSDH remain controversial, and treatment options, including surgical evacuation, are still debatable. Despite being one of the oldest and simplest neurosurgical procedures, establishing clear recommendations or guidelines on CSDH management may remain a challenging task for the time being.

We hope this Research Topic inspires further research and discussion within the neurology and neurosurgery communities, ultimately leading to enhanced care for CSDH patients.

## Author contributions

ML: Writing – original draft, Writing – review & editing. HS: Writing – original draft, Writing – review & editing.
